# Odour and indoor air quality hazards in railway cars: an Australian mixed methods case study

**DOI:** 10.1007/s40201-024-00908-y

**Published:** 2024-06-24

**Authors:** Shaneel Chandra, Lisa Bricknell, Sandrine Makiela, Sherie Bruce, Anjum Naweed

**Affiliations:** 1 College of Science and Sustainability, School of Health, Medical and Applied Sciences, Central Queensland University, Rockhampton North, QLD 4702 Australia; 2 Appleton Institute for Behavioural Science, Central Queensland University, 44 Greenhill Road, Wayville, SA 5034 Australia

**Keywords:** Public health, Railways, Risk, Indoor air quality, Odour

## Abstract

**Purpose:**

This case study aimed to diagnose the cause(s) of a seasonal, and objectionable odour reported by travellers and drivers in the railway cars of Australian passenger trains. The research questions were to: (1) identify whether significant microbial colonisation was present within the air handling system of trains and causing the odours; to (2) identify other potential sources and; (3) remedial options for addressing the issue.

**Methods:**

A mixed-methods, action research design was used adopted. Sections of the heating, ventilation, and air conditioning (HVAC) systems from odour-affected trains were swabbed for bacteria and fungi and examined for evidence of wear, fatigue and damage on-site and off-site. Insulation foam material extracted from the walls of affected trains was also subjected to a chemical assessment following exposure to varying humidity and temperature conditions in a climate simulator. This was accompanied by a qualitative sensory characterisation.

**Results:**

Upon exposure to a variety of simulated temperature and humidity combinations to recreate the odour, volatile chemical compounds released from the insulation foam by water were identified as its likely cause. In addition, a range of potentially serious pathogenic and odour-causing microbes were cultured from the HVAC systems, although it is considered unlikely that bacterial colonies were the odour source.

**Conclusion:**

The research has implications for the sanitising and maintenance policies for HVAC systems on public transport, especially when operating in humid environments. The sanitary imposition, especially in the wake of COVID-19 may be required to ensure the safety of the travelling public and drivers.

## Introduction

Indoor air quality is often compromised as a result of unpleasant odours, particularly within enclosed spaces such as automobiles [1, 2], public transport, and the built environment in general [3]. Microbial growth in air handling systems has been implicated as the source of such odours [4], often described as musty, sour, noxious, and similar to body odour and rotting fish [5]. Any such odour in an enclosed space tends to cause discomfort, and this can be translated by the public as hazardous to their health, even in the absence of pathogenic effects [6, 7].

Apart from the psychosomatic health issues relating to odour, microbial contamination of air handling systems have been widely implicated as the source of pathogenic infection, and linked to cases and outbreaks of legionellosis, aspergillosis, SARS, influenza, measles and tuberculosis [8–10]. More recently, air handling systems have also been implicated in the transmission of the SARS-CoV-2 virus during the COVID-19 pandemic [10, 11]. To prevent health risks, exposure to harmful materials such as particulate matter in public transport ventilation systems must be kept to a minimum, and the traveling public tends to be cognisant of this [12].

Modern air handling systems are often large and complex, and effective cleaning and disinfection can be difficult to achieve [13]. Research has shown that colonisation of evaporator fins by odour-causing microbes is common [4, 14]. Fungal colonies grow well on insulation materials which absorb moisture [15], and at least one outbreak of disease has been linked to degraded insulation in an air handling system contaminated with *Aspergillus spp* [9]. Once microbial colonies have established themselves in air handling systems, the likelihood of them becoming a source of adverse air pollution of microbial origin will grow the longer it is used [16].

Other odours in enclosed spaces can be attributed to chemical sources. Synthetic materials used as adhesives, furnishings, insulation and paint are known to release volatile organic compounds (VOCs) such as benzene (associated with leather) [17] and formaldehyde (in new products generally) [18] that are odourous, some of which contribute to the characteristic “new car smell” [19–22]. Examples include aromatic hydrocarbons, terpenes and a large variety of aliphatic and cyclic hydrocarbons as well as carbonyl compounds, including formaldehyde [22]. Other odours are known to occur as the result of chemical reactions that form secondary pollutants [21, 23]. The range of odours of purely chemical origin is substantial, and dependent on the compound; for example, methyl mercaptan produces an odour of rotten cabbage while η-butyl acetate, a component of some paints and varnishes, has the scent of ripe bananas [24].

While odour sensation depends on the concentration of molecules in the air, individual recognition of a culpable compound is often difficult because odours blend as a result of the complex mixture of chemicals in indoor environments [24]. This is exacerbated by the infinite variation in human anatomy, physiology, and genetic makeup, as well as familiarity and cultural background, all of which play a role in how humans perceive odours [6, 7, 24–27].

### Research context & aims

In early 2020, reports emerged of intermittent but persistent odour complaints over the preceding 12 months in the passenger and driver cabins of new trains in a large metro area in Australia [28]. These trains were part of a recently commissioned stock. The odour was reported more in the summer-autumn periods of high temperature and high humidity and particularly noticeable when train air conditioners operated on ventilation and cooling modes. Descriptions of the odour ranged from ‘cat urine’ or ‘sweaty socks’ with agreement that it was strongly apparent within the first 30 min of operating the air conditioners and tended to dissipate over several hours. Subsequent measures to tackle the odour included in-house physical cleaning of externally accessible components of the air conditioning (hereafter referred to as the HVAC– heating, ventilation, and air conditioning) unit and placement of various odour masking agents. These were ineffective. Moreover, commercial measurements, screening and analysis (volatile organic compound levels, condensate water from air conditioning, surface samples) did not identify a probable cause or rectify the problem. This paper presents an empirical investigation that was undertaken to identify the cause(s) of odours on these new trains and subsequent mitigation and treatment measures to help address the issue. Although research has examined air quality in indoor and outdoor environments, this study is the first to report it scientifically in the context of public transport with an environmental origin. Given the relationship between objectionable odour and air handling systems [4, 14, 29], the aims of this research were threefold:

1. Investigate implicated rail cars to determine if significant microbial colonisation was present within the air handling system;
2. Determine the likelihood that microbial contamination was the cause of the objectionable odours; and.
3. Identify other potential sources of the odour.

## Methodology

### Research design

**Fig. 1:**
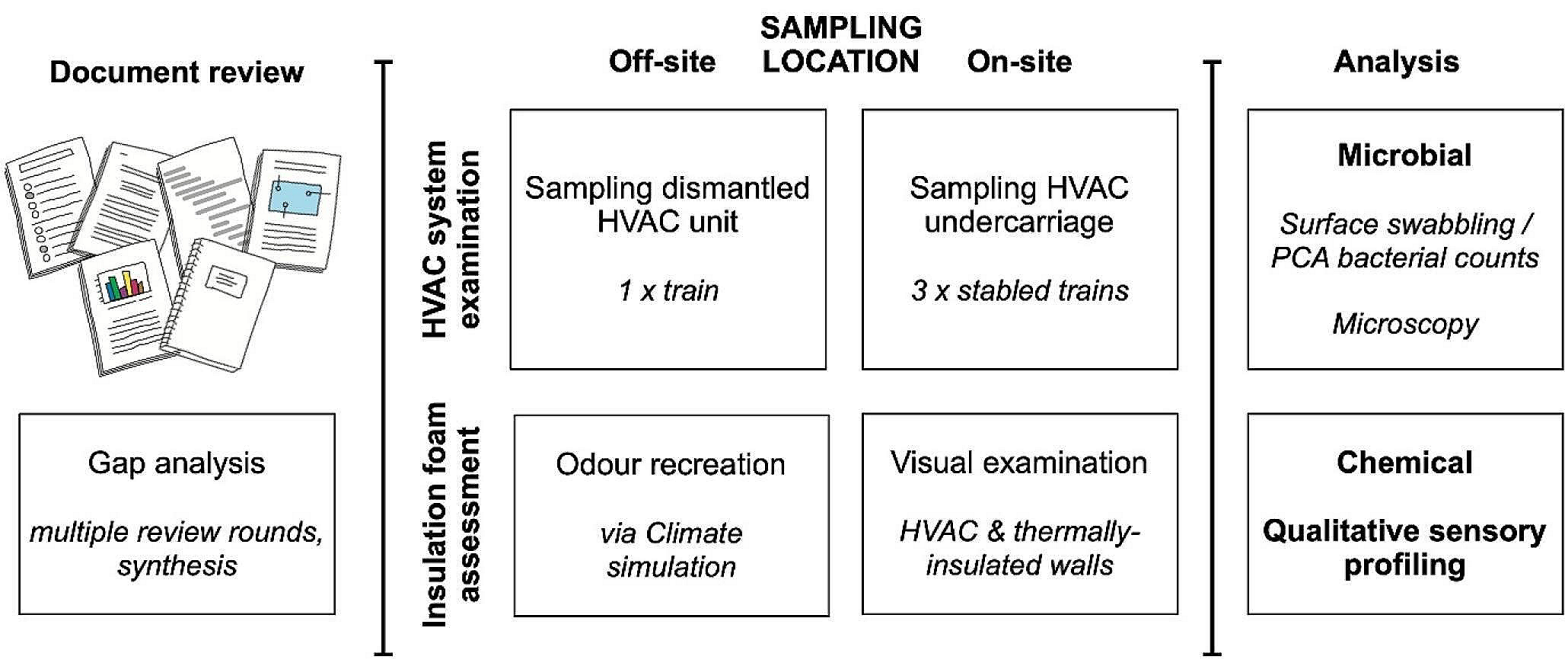
Overall research design and study methodology. Figure created using OmniGraffle (7.2.2.2)

A mixed-methods action-research design utilising a case study approach was adopted [e.g., 30, 31, 32] where success or failure of experimentation enabled exclusion or inclusion of causes. This allowed the research to be progressed iteratively in parallel with literature search, artefact analysis and scientific sampling and analysis. Given the nature of this study and topic, an interdisciplinary team was assembled comprising senior research expertise in environmental health science, transport human factors, microbiology, and chemistry. The investigative process used a bifurcated approach, initially investigating the hypothesis from the manufacturer that the odour was originating from the HVAC system, and later investigating the insulation in walls and floor spaces. Both biological and chemical causes were considered in a procedure that also included initial document review. The process is illustrated in Fig. 1.

### Procedure

#### Document review

A wide range and variety of reports and memo content was made available to the team by the manufacturer. These included cleaning manuals and datasheets, preliminary problem-solving processes and root cause analyses, commercial laboratory testing reports, preliminary assessments, and monitoring plans. These were reviewed using a traditional critical literature review approach supplemented with a gap analysis. Several frameworks for gap analysis exist, and here, a four-step approach used was, as follows:

1. The content in each supplied document was read by four researchers, with group discussion around initial impressions and points that were particularly striking;
2. Documents were individually reviewed by four researchers, noting key points, observations, and notation of overriding (research/logic) questions; substantive empirical studies and literature were sourced to direct and providing underpinning support where needed;
3. The individual analyses were collated and synthesised into an overarching structure, with gaps identified based on study aims (identification of cause and assessment of prospective mitigation). These were based on: (a) the current status; (b) the desired or ideal (future) state; (c) description and identification of the gap(s); and (d) remedial action(s); and.
4. Ongoing discussions were held to critique and refine individual the gaps and find consensus.

#### Physical examination: on-site and off-site

During the second quarter of 2020, restrictions on non-critical human movement due to the global pandemic precluded researchers from visiting and observing trains during their operation. Thus, experiencing the odour phenomenon *in situ* was not possible. Moreover, the odour was an outcome that only occurred when a particular combination of season, climatic conditions, time, routes, and carriages was achieved, and thus was not able to be recreated to suit a systematic methodology. These factors prevented a strip down of the entire carriage and comprehensive follow-up analysis of every component, and encouraged adoption of a deductive approach targeting the components most likely to be the source. Therefore, the manufacturer organised the expedited extraction and delivery of a HVAC unit from a train known to be affected by the odour directly to the researchers’ location. The HVAC unit was couriered overnight and stored in an enclosed shed. A technician skilled in air conditioning and HVAC system servicing travelled to the research facility to assist the researchers with their queries. The HVAC unit was systematically dismantled by the technician in order to grant researchers access to required sections. These were swabbed for bacteria and fungi and examined for evidence of wear, fatigue and damage.

A month following offsite examination, researchers were able to travel to the manufacturer’s stabling facility and access trains for on-site sampling. Three trains were accessed and the passenger-facing undercarriage of their HVAC systems (inaccessible during offsite examination) were examined. Train maintenance and service activities being undertaken were also observed.

### Analysis

#### Microbial analysis

**Fig. 2:**
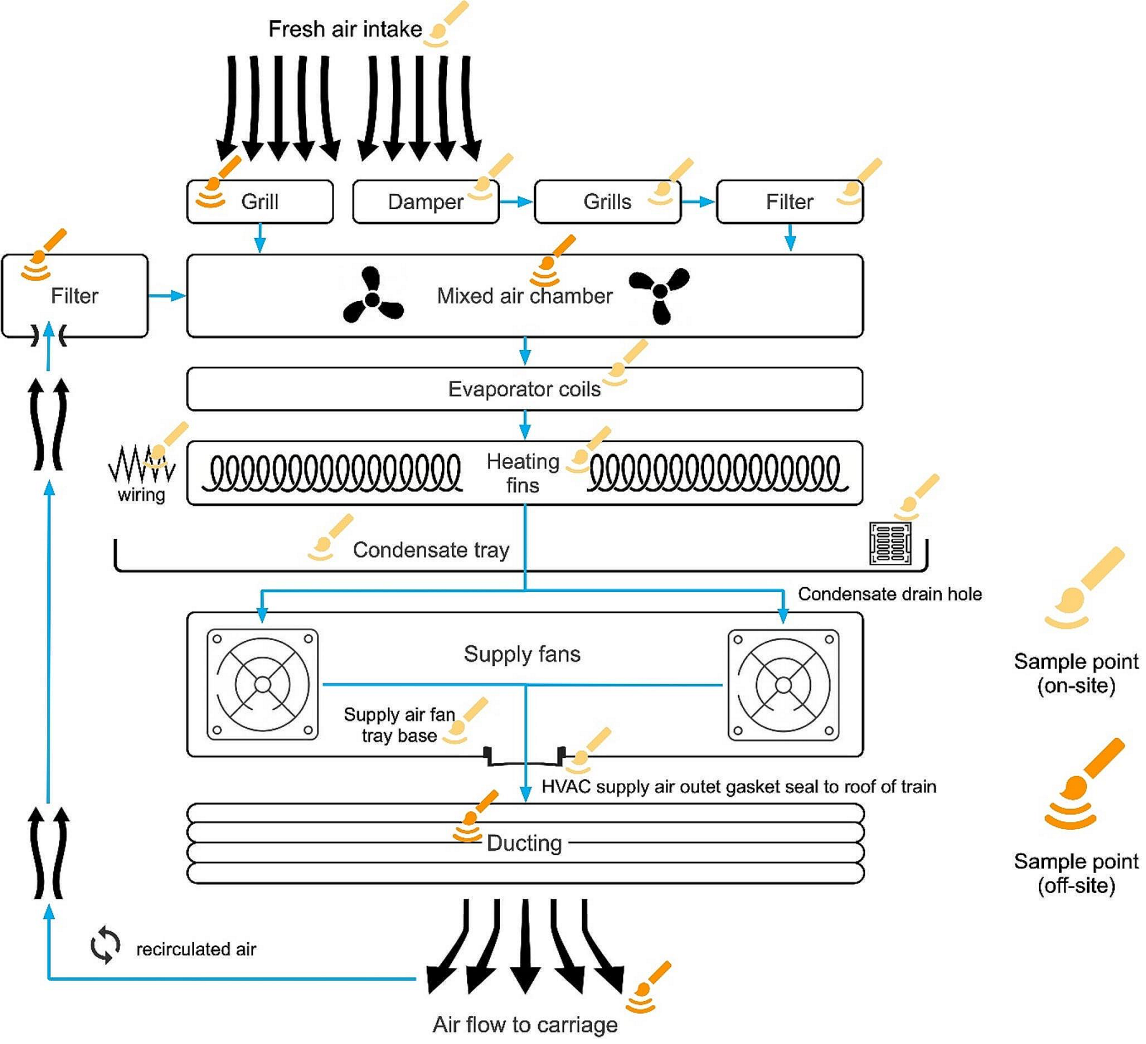
HVAC schematic showing specific sample locations for microbial analysis and where they were taken (on-site, off-site). Figure created using OmniGraffle (7.2.2.2)

Figure 2 shows an overall schematic of the HVAC system used on the trains with specific sampling locations for microbial analysis. A series of components (damper, grill, filter) were part of the fresh-air intake process prior to mixture with separated filtered recirculated air. Swabs were taken within the HVAC unit and the distribution ducting in the train’s ceiling. A dry swab was wiped in a standard cross hatched pattern against the surface in a 50 mm x 50 mm template. Five replicate samples were collected from each of the 16 sites (80 samples) for microbial analysis. All swabs were transported under protocol for microbial and fungal examination at the university’s laboratories.

The microbes collected during sampling were cultured on several types of growth media allowing for a broad differentiation of the type of microbe. These included Plate Count Agar (PCA), Tryptic Soy Agar (TSA), Potato Dextrose Agar (PDA), and Sabouraud Dextrose Agar (SDA). The PCA medium is a non-specific and non-differential agar used to grow a broad spectrum of microbes where counts refer to the total number of bacteria found in the sample. TSA is another general-purpose medium that tends to be favoured by bacteria and is used to isolate bacteria for more specific identification [33]. In this study, TSA counts were related to bacteria associated with humans. PDA is a medium specifically used to cultivate fungi [34] that encourages mould sporulation and pigment production in a number of species associated with skin, which assists in identification. Lastly, SBA is a growth medium that is used for the cultivation and differentiation of fungal species, particularly those that grow on skin [35].

One sample from each site (16) was transported to the Australian Centre for Ecogenomics at the University of Queensland for genetic sequencing to identify the spectrum of genera colonising the system.

#### Chemical assessment

Samples of insulation foam from affected trains were obtained from which subsamples (*N* = 4) of 10 cm (depth) × 10 cm (width) × 15 cm (length) were collected and subjected to a range of weather parameters (temperature, humidity and moisture) in an enclosed, isolated climate simulator. The simulator was able to replicate conditions for the spring and summer seasons when complaints were received. Subsamples were placed inside the simulator and exposed for 30 min. During this period, the temperature and relative humidity were continually monitored. After 30 min, the insulation materials were placed inside a drying oven (Memmert UM-400– GmbH + Co.) for a period of 3 h, with moderate air flow. To avoid cross-contamination, the insulation samples were placed in the oven from the same weather simulation experimental conditions.

The chemical assessment sought to determine whether the humidification and drying cycles induced chemical changes at the foam outer surface. A staple test in such determinations is changes to the surface pH. Thus, to measure the pH of the foam sample surface before and after the drying stage, a strip of semi-quantitative litmus paper was placed atop the foam for 10 min after the foam was removed from the simulator. Next, the litmus paper was examined against the graduation scale for pH measurement. The process was repeated for the last 10 min of the drying stage. The strip was examined after the foam was removed and the corresponding pH was documented. In this manner, the changes in pH at the foam surface before and after the drying stage could be compared.

Following the drying period, the foam was subjected to storage in a dry, sealed plastic box for descriptive sensory characterisation.

#### Descriptive sensory characterisation

An evaluation was conducted on the odour produced from the insulation material after it had dried following exposure to simulated climatic conditions in the simulator. This was a qualitative descriptive sensory characterisation, and the odour was evaluated using a set of variables including odour intensity, hedonic tone and odour note [6, 20, 21, 23, 36–38].

Eight individuals of the extended research team (key researchers and additional laboratory/academic staff involved in the project) participated in the evaluation by independently characterising the odour on the insulation material. This took place in a well-ventilated laboratory on university premises in a space free from use of solvents or other odorous compounds.

Each individual was asked to open a foil package containing a sample of the treated insulation material and, using their hand, waft air over the material toward their nose. They were then asked to complete a short questionnaire, characterising any odour they may have perceived, or identifying if there was no perceptible odour.

Odour intensity was assessed using two variables: *strength* and *pervasiveness*, both on a categorical scale analogous to a Likert scale. The strength of the odour could be categorised as: very strong, strong, faint but noticeable, barely noticeable, or undetectable. The pervasiveness of the odour was assessed on a similar scale: very pervasive, pervasive, less pervasive or not pervasive. Pervasiveness was clarified by the subtext explanation: *an odour may be very faint but be quite pervasive and annoying, causing headaches, sneezing or distraction*.

Hedonic tone was assessed by asking the participant to identify the odour as pleasant or unpleasant.

**Fig. 3:**
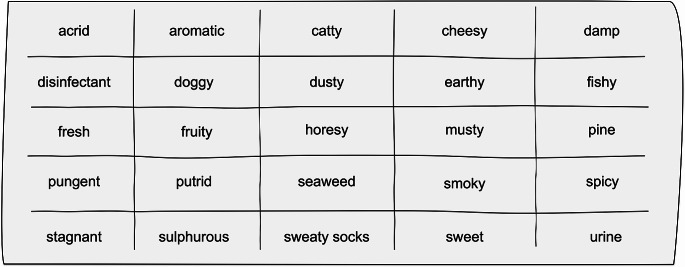
Alphabetised list of adjectives used in the descriptive sensory characterisation. Figure created using OmniGraffle (7.2.2.2)

Finally, odour note was assessed using two variables. The first question asked individuals to describe the odour in their own words. The second question, presented subsequently, asked individuals to select words describing the odour from a pre-defined set of 25 adjectives. The adjectives were compiled into a grid and included descriptions in complaints/reports received from commuters as well as a variety selected from similar studies (Fig. 3).

As the relationship between odour and concentration is contentious [36, 39], this exercise did not aim to provide quantifiable data relating to an estimation of the concentration of chemical species or the actual strength of the odour. Instead, it was designed to provide a qualitative characterisation of the odour only, in effort to help determine if the odour produced in the laboratory via the climate simulator was the same as that reported by complainants.

## Results

### Document review

Complainant reports contained descriptions of the objectional odour on trains as sweaty socks, fishy, musty and earthy. The document review identified that investigations conducted by occupational hygiene consultants had not identified any volatile organic compound likely to produce an odour and air sampling had not contained any bacteria or spores associated with similar odours.

The cleaning of the evaporator coils of HVAC units in affected trains had elicited some improvement, suggesting that microbial colonisation of the air handling system could be the source of the odour. A scoping review of literature supported this theory, showing that bacterial genera such as *Methylobacter* and *Staphylococcus* are responsible for similar odours and have been known to colonise HVAC systems [4, 14, 29].

Document review and discussions with the manufacturer raised a secondary hypothesis that the odour was chemical in origin, specifically from isothiazole ring-based chemicals incorporated into paints used in construction or from insulation material that has been allowed to become wet. These compounds are added to marine paints and anti-fouling agents and also to paints to prevent mould colonisation. Previous studies have shown that isothiazole compounds can off-gas from water-based paints and associate with a pyridine odour [40, 41]. An odour associated with these substances, often pyridine, has sometimes described as *“fishy.”* A characteristic urine-like odour associated with ammonia or amines such as trimethylamine (TMA) have also been identified [42]. Complaints described the objectional odour in various terms, such as *“sweaty socks”, “fishy”, “musty,” “earthy”* and *“urine”* thought it was also clear that not all could smell the odour. Documents reviewed indicated that significant volumes of water were collected in the substructure of the train carriages, although the source was uncertain.

### HVAC system examination– off-site

Table 1 shows the results of the off-site microbial analysis conducted on the HVAC system. Evidence was found of soiling and microbial activity on the HVAC rooftop and on the tray base surrounding the supply air fans. In addition, the flexible gasket seal on the underside of the unit (through which conditioned air flows in the carriage ducting) was damaged through what appeared to be routine mechanical wear. Organic-origin material was observed in the condensate drain located below the heating coils—likely intrusion points for fungal and bacterial presence. Cultures grown from swabs of these areas all revealed the presence of both fungi and bacteria. This was to be expected; however, fungal loads were particularly high around the supply air fan, heater coil wiring and the return air grill. Bacterial counts were reasonably low, apart from samples taken from the area surround the gasket through which cooled air is vented into the passenger compartment.

**Table 1:** Microbial counts found in HVAC system in the off-site physical examination

Location and description	Bacteria counts		Fungi counts	
	PCA	TSA	PDA	SAB
Fresh air intake	7	21	18	32
Fres air damper	6	2	4	7
Fresh air grill (i.e., pre-filter)	34	43	91	86
Fresh air filter	4	1	61	90
Evaporator coils	19	19	2	4
Heating fins wiring	4	2	119	> 200
Heating fins	58	57	> 200	> 200
Condensate drain hole	1	41	39	21
Condensate tray	62	9	32	14
Supply air fan tray base	24	10	> 200	156
HVAC supply air outlet gasket	206	132	48	18

While bacteria were found in significant numbers throughout the HVAC unit, reported counts only reflected those able to be cultured on the media; thus it is likely, and somewhat concerning, that bacterial counts would be higher overall. There were also high fungal counts around the fresh air grills, the tray surrounding the supply air fans and wiring adjacent to the heating coils.

Table 2 illustrates the full spectrum of genera identified by genetic sequencing. Many of the genera identified match those commonly identified in other research relating to air cooled spaces, including *Methylobacter*, *Staphylococcus, Bacillus, Klebsiella, Enterobacter, Streptococcus and Corynebacterium* [4, 14, 29, 43–46]. *Methylobacter spp* and *Staphylococcus spp* were found to be present, and are recognised as producing unpleasant odours associated with air conditioning systems [4, 14, 15, 29, 44–46].

**Table 2:** Spectrum of genera identified through genomic sequencing

Genus	Species
*Bacillus*	*B. anthracis*
	*B. cereus*
	*B. smithii*
	Other uncultured *B. spp*.
*Bifidobacterium*	Various uncultured *Bif. spp*.
*Clostridium*	*Cl. botulinum*
	*Cl. perfringens*
	Other uncultured *Cl. spp.*
*Corynebacterium*	Various uncultured *Coryne. spp*.
*Enterobacter*	Various uncultured *Enterobacter spp*.
*Enterococcus*	Various uncultured *Enterococcus* spp.
*Haemophilus*	Various uncultured *H. spp*.
*Klebsiella*	*Kl. pneumoniae*
	Other uncultured *Kl. spp*.
*Lactobacillus*	Various uncultured *Lact. spp*.
*Legionella*	Various uncultured *Leg. spp*.
*Methylobacterium- Methylorubrum*	Various uncultured *Methyl. spp*.
*Mycobacterium*	Various uncultured *Myc. spp*.
*Neisseria*	Various uncultured *N. spp*.
*Pseudomonas*	Various uncultured *Ps. spp*.
*Rickettsia*	Various uncultured *R. spp*.
*Serratia*	*Ser. marcescens*
*Staphylococcus*	Various uncultured *Staph. spp.*
*Streptococcus*	Various uncultured *Strep. spp*.

**Table 3:** Microbial counts for swabs taken during on-site examination

Count	Return air filter	Mixed air chamber	Filter	Ducting 1	Ducting 2
Bacteria	22	9	74	48	146
Fungi	6	11	15	> 200	> 200

### HVAC system examination– on-site

**Fig. 4:**
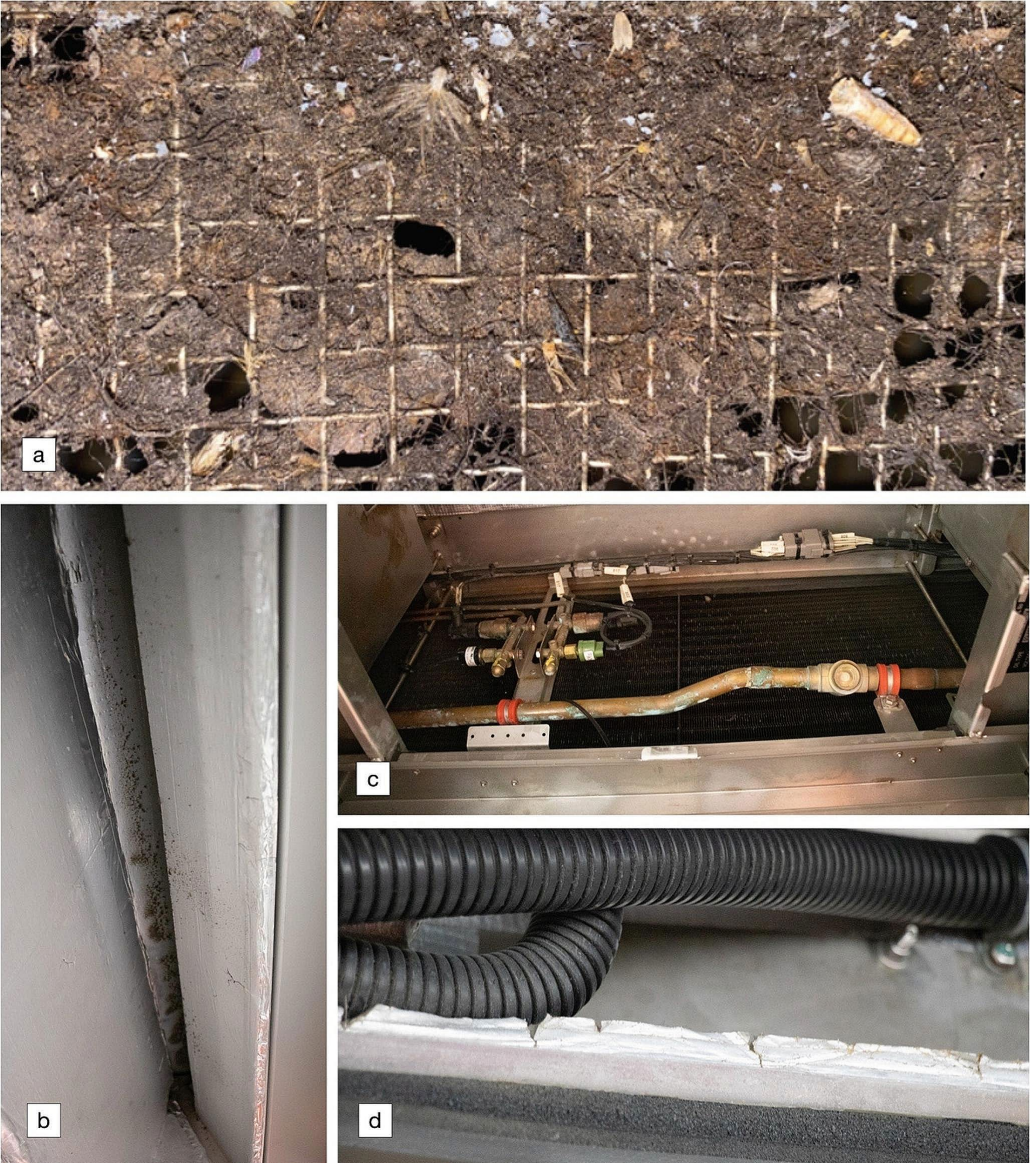
Photos taken of the HVAC unit of an odour-affected train during the off-site examination showing: (**a**) organic build-up on the fresh air intake grill, (**b**) microbial growth inside ducting, (**c**) corrosion in the piping, and (**d**) damaged insulation foam seal

During on-site examination of the HVAC system, focus was on components inaccessible to researchers during the off-site examination. Findings showed that the return air filter was severely soiled with dirt, detritus, and dead insect bodies. No odour was encountered during the train’s stabling period; however, the foam sealant was found to be damaged, potentially causing water intrusion, and thus a site for bacterial growth. As shown in Fig. 4, there was also evidence of corrosion within the HVAC unit.

While this train was brought in for routine, 3-monthly service, the extent of degeneration and soiling of the components indicated that the three-monthly service either did not extend to ensuring their longevity or, exceeded their fatigue boundaries altogether. Observation of the storage facilities revealed that some vital components of HVAC systems, such as the air filters, were not stored away from atmospheric elements.

Table 3 shows the microbial counts for swabs that were taken at the on-site examination. As in the off-site examine, these were limited to organisms able to grow on artificial media, so actual counts were likely much higher. Counts were similar to those of off-site examination, and similar species were found (e.g., *Staphylococcus*). The ducting was of concern, as such high fungal numbers could easily trigger allergic responses.

### Insulation foam material assessment

**Fig. 5:**
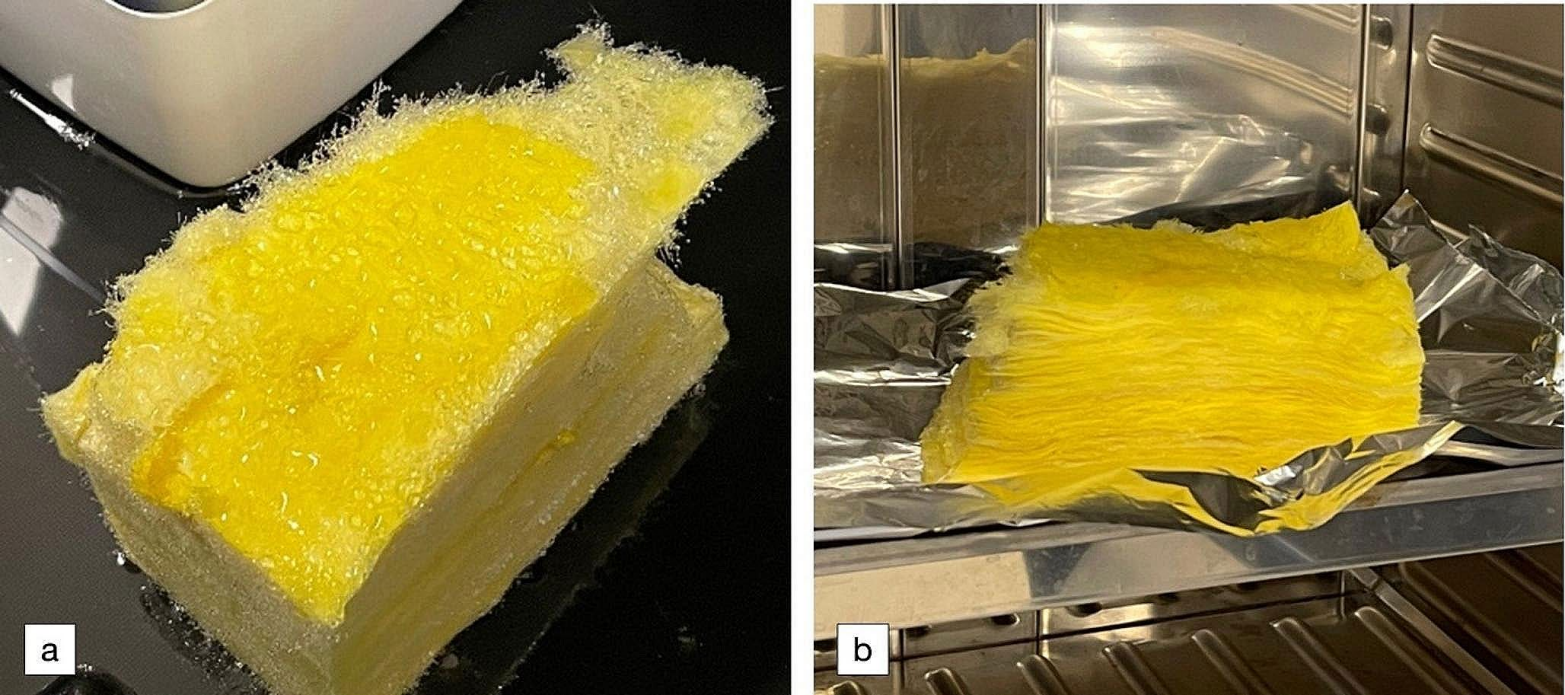
Photo of a sample of insulation foam shown (**a**) inside the climate simulator after an experiment, and (**b**) another section inside the heating oven

No particular combination and/or adjustment of temperature or humidity setting on the climate simulator elicited any odour issues from the insulation material. Figure 5(a) shows the insulation foam material after being placed inside the climate simulator. The presence of spherical droplets of water indicates a high degree of surface hydrophobicity on the material surface. As such, strong hydrophobicity may have limited the penetration and absorption of moisture inside its layers. However, some ingress of water within the layered sheets comprising the material was observed. Thus, while there was not much wetting of the material bulk to speak of, interstitial water ingress between the foam material layers and crystals would have reasonably led to some chemical interaction at the water/solid interface.

Figure 5(b) shows the insulation foam material placed in the drying oven. The drying period naturally evaporated off the water droplets from the material’s outer surface, but a very strong odour also manifested inside the oven which was not previously evident. This suggested that the drying out of the material released the volatile components from aqueous to gas phase on the material’s surface.

Prior to simulation in the climate simulator, the pH of the insulation foam material surface was close to 6.0, representing mild acidity potentially imparted by slightly acidic chemical compounds on the outer surface. Following the heat and dry stages, an unidentified physicochemical transformation likely occurred on the outer surface of the foam sample, causing the release of odorous chemical species. While no chemical fingerprinting was undertaken, the pH was observed to have increased to 8.0 after drying off the water. As an increase of 2 pH units was a significant change in the chemistry of the surface, this suggested that the compounds causing the odour and basicity may have had strong potency and/or exist in high concentrations.

### Qualitative sensory profiling

When exposed to the treated insulation, seven individuals (i.e., all but one) reported being able to smell an odour. Most considered the odour to be strong to very strong, and all who could detect the odour considered it to be somewhat pervasive. All participants who were able to detect the odour also reported finding it unpleasant.

When asked to self-describe the odour, descriptions were mixed, but common words were used. The most common word/analogy adopted was “urine” (five individuals) with one expanding this description to *“like a cat’s litterbox”.* Another individual described the odour as *“eye watering”* and *“like a day’s old nappy soaked with urine”.* Other common responses were *“musty”* or *“fishy”*, and one individual described it as having *“echoes of rubber and fish.”*

When provided with the sheet of adjectives describing odours (see Fig. 3), “urine” was again the most commonly identified adjective. This was followed by *“fishy”, “pungent”, “musty”* and “*sweaty socks.”* Terms like *“fishy”, “urine”, “pungent”* and *“cat’s litterbox”* are associated with the presence of amines like trimethylamine for fishy odours [47], terpenes for musty odours [48] and amides for pungent odours [49], all of which tend to be both basic and odorous.

Taken together, the results resonated with complainant reports of affected trains. Given that TMA has a characteristic urine-like odour [42] and is often associated with insulation materials exposed to significant amounts of water [50], TMA emerged as the likeliest candidate for the objectionable odour in the affected trains.

## Discussion

In broad terms, this study sought to identify cause(s) of seasonal odours in passenger trains but, noting the environmental health implications associated with the specific research context, its aims were to: (1) determine if significant microbial colonisation was present within the HVAC system of affected trains, (2) determine the likelihood of microbial contamination being the cause of objectionable odours, and (3) identify other potential sources of such odours. Based on the reiterative nature of the odour phenomenon, odours were originally hypothesised to be bacterial in origin. Although several species of odour causing bacteria were identified in HVAC system components, colonies were not sufficient to explain widespread reports of objectionable odour across several different trains. On this basis, the hypothesis of bacterial causes for the odours could be rejected. The next step was to identify other potential sources of the odour. An examination of the carriage layout and detailed verification with the train manufacturers did not identify any specific potential site or section of the affected carriages that could have been the source, such as toilets or soft furnishings. Additionally, because the occurrence was across several different trains over an extended period of time, the cause could not reasonably be attributed to individual passengers and needed to be something systemic.

The condition of HVAC components was a concerning discovery, given that some were at the point of fatigue. Failing seals were clear pathways for water intrusion, especially on rainy days when trains are in motion. Water permeating through these barriers would inevitably lead to its ingress in areas where moisture prevention is critical, such as onto seats, and importantly, in the panelling, and thus the insulation. With the advanced stage of degeneration of the seals, sustained moisture intrusion was almost certain. Evidence from the literature identifies that odours are known to occur when insulation material is exposed to moisture [50], which led to the hypothesis that the insulation material, in the presence of moisture, was the source of the odour.

### What was the odour and was it a health issue?

The simulation process appeared to produce TMA, but whether the odour was the same as the one reported by complainants during the summer of 2019–2020 is difficult to substantiate. TMA is known for its fishy odour at low concentrations (below 100 ppm) [41, 47, 50, 51]. At air concentrations greater than 100 ppm, the odour is almost indistinguishable from ammonia [47, 51]. This corresponded with sensory profiling and given the frequency with which the “urine” descriptor was used, it was very possible that concentrations exceeded the 100 ppm threshold for some individuals, while others were exposed to lower levels. The correlation in the description of the odour strongly suggested that the odour produced in the laboratory was related to volatilised TMA or other, similar, methylamine compounds. Reports from complaints described the odour in various terms. The variation in detection and strength of the odour also matched the study and is not unusual; the differences in olfactory perception between individuals is well established [26, 39, 52]. While it is not possible to determine without doubt that the odour produced via simulation was the same, the similarity in conditions and description makes it likely.

If the odour of affected trains was indeed TMA, it is unlikely that levels of TMA were high enough to present a health issue for passengers. The USEPA interim Acute Exposure Guideline Levels (AEGL) for TMA set Level of Distinct Odour Awareness (LOA) of 0.00051 ppm in their AEGL Technical Support Document [53]. At this level, effects are not disabling, temporary, and resolve after exposure cessation. The AEGL-1 for TMA was derived using human data by the American Industrial Hygiene Association and set at 8.0 ppm. If those reporting an odour similar to ammonia experienced concentrations above the 100 ppm proposed by Deichman and Gerarde [54], this would relate to an exposure that caused transient discomfort and irritation.

At levels falling within the LOA range of 0.00051 ppm and the 30-minute AEGL-2 level of 150 ppm, the odour would be described as akin to ammonia but produce more severe respiratory effects [53]. As these more serious effects were not reported, it is assumed that the concentrations were high enough to cause an ammonia-like odour at times, but not high enough to cause significant health effects, and instead, were transitory and non-disabling. This would suggest that the risk of TMA exposure to passengers was low. However, given that TMA exposure does carry a toxicological risk, it would be prudent to implement control strategies if workers were tasked with removing the affected insulation.

### Pathogenic risks to staff and commuters using passenger trains

During investigation of the source of the odour, microbial swabs identified a range of different microbial species, many being recognised agents of disease. Although the goal of our investigation was to identify the source of the odour, these colonies demonstrate an additional, potentially more serious hazard, to staff and commuters.

As the association between microbial growth with moisture is well-established [55], the infiltrated moisture inside the train superstructure is very likely to have contributed to microbial growth within the trains. While the cause of the odours appeared to be unrelated to bacteria and fungi presence, there remains some risk of human exposure to pathogens, some more serious than others. Given the range of genera identified that have the potential to be pathogenic to humans, and the high microbial counts, a more frequent and robust cleaning regime is clearly a requirement to address a potential outbreak more serious than uncomfortable odours.

A number of individual species and genera were identified by the genomic sequencing that are considered human pathogens: *Bacillus anthracis* is the infectious agent for anthrax [56]; *Clostridium botulinium* is associated with the production of a group of neurotoxins called botuliniums that cause muscular paralysis– an association so potent that the species is even considered to be a bioterrorism agent [57]; *Klebsiella pneumoniae* has a potential to cause pneumonia and is resistant to carbapenems, which are usually the antibiotics of the last resort [58]; *Legionella spp.* cause legionellosis, and is a serious and potentially fatal disease especially for older (> 50 years of age), debilitated, and immunocompromised people [59]; *Streptococcus spp.* is responsible for a range of common respiratory and skin infections but are also responsible for invasive bacterial infections including sepsis [60]; and *Ser. marcescens*, while widespread in the environment, is also a cause of a range of infections of the blood, respiratory system and gastrointestinal tract [61]—it is of concern to immunocompromised patients [62] and in the hospital setting [63] particularly in neonatal intensive care, as it is intrinsically resistant to several antibiotic classes [61]. In short, almost all the different bacteria known to cause most hospital acquired infections were identified in the HVAC system in the off-site examination.

Based on the on-site observations at the train stabling facility, HVAC components such as air filters were not stored away from the atmospheric elements and therefore exposed to prolonged moisture and airborne particulates. Air filters are particularly sensitive components that need proper storage. The exposure to long periods of high relative humidity (> 80%) causes a proliferation of bacteria on air filters with subsequent release into the filtered air and inclusion into the respirable fraction [64]. The relative humidity in the area is known to range between 75 − 77% over the summer [65], hence approaching the critical moisture levels in the air for microbial growth to have proliferated. These filters therefore presented an opportunity to introduce microbial cultures into the air handling system and, once introduced, colonise any environment with suitable conditions for growth, such as moisture and protein from skin cells and insect debris.

It must be acknowledged that the risk presented by these bacterial colonies was likely low, although it was difficult to assess it accurately given the difficulty establishing a dose response relationship between airborne bacterial concentrations and disease [66, 67]. What the results did identify, however, is the existence of a microbial hazard that was not currently being addressed as part of routine maintenance activities.

### Implications for cleaning, sanitising and maintenance of HVAC systems on public transport

While this study focused on metropolitan trains, it has implications for all public transport utilities with HVAC systems operating in similar humid environments. As reflected in our findings, an oversight into equipment maintenance or material fatigue can lead to intrusion of moisture in components improperly equipped to handle it in a safe manner. As a result, there are flow-on consequences such as microbial growth which can lead to health problems for persons, especially with pre-existing conditions breathing air from such HVAC systems. While our investigation did not establish microbial contamination of the HVAC system as a cause of odour, research during the COVID-19 pandemic has further established that air handling systems in enclosed spaces are potential sources of exposure to pathogens [10] and that maintaining a high standard of indoor air quality is crucial to public health. A greater awareness of these risks should lead to continuous updating of standard operating procedures, for example those around HVAC servicing to keep ahead of potential health-related problems. This is more so in present times where air recycling is implicated as a vector for spreading dangerous particles like the COVID-19 viral variants that can easily escape fine filters. The transportation industry could therefore benefit from a strategic rethink around the design, operation and maintenance aspects to prevent emerging problems. This should be accompanied by public education as part of health promotion around the risks of public transportation use with enclosed, recycled, shared air. Core to this education initiative would be the inclusion of train frontline workers who would be the most exposed to any dangers from the air coming out of the HVAC systems.

Trains are recognised as potential environments for disease transmission. The European Union’s COVID-19 Rail Protocol addresses train cleaning and disinfection, recommended passenger behaviour, measures to increase natural ventilation, and equipment upgrades to prevent COVID-19 transmission [68]. The protocol provides specific requirements that cleaning of HVAC systems be performed according to manufacturer’s instructions [68].

In Australia, the Department of Climate Change and Energy Efficiency has a best practice maintenance and operation guideline for HVACs, but this is purely from an energy efficiency perspective [69]. The NSW Transport Assets Standards Authority [70] have published a Standard for Climate Comfort and HVAC on Passenger Rolling Stock that specifies that air in trains must be substantially free from “dust, contaminants and impurities” and must comply with Safe Work Australia’s Workplace Exposure Standards for Airborne Contaminants. However, neither documents include requirements for regular cleaning or disinfection of air handling systems [70, 71]. Australian Standard AS7402:2022 addresses design issues for HVAC systems used in trains, specifically identifying the need for their design to facilitate easy cleaning of condenser and evaporator coils, condensate drains and pans, and rainwater drains [72]. Currently, no industry documents within Australia address the necessity for, or frequency of, cleaning and sanitising of HVAC systems in public transport. From this, one may infer an expectation that manufacturer instructions are to be followed with respect to cleaning and disinfection. Without this, cleaning of the system may only take place when air quality exceeds Safe Work Workplace Exposure Standards. The issue here, then, is the absence of mandated air quality monitoring in trains, meaning that a requirement for cleaning and disinfection would only be triggered upon complaint or evidence of disease transmission.

An absence of freely available documentation around cleaning and disinfection of HVAC in public transport suggests that the reliance on transport companies to follow manufacturer’s recommendations is widespread. Given the implication of air handling systems in the transmission of respiratory diseases and the nature of risk exposure in public transportation, this presents a hazard to all service workers and commuters alike. With some nations adopting requirements for routine sanitising procedures for trains, it may be advantageous for jurisdictions in Australia and abroad to follow suit with mandated maintenance policies for HVAC systems on public transport.

### Strengths & limitations

This study was conducted with an applied, action research design orientation, meaning it aimed to simultaneously investigate and solve an issue relatively flexibly and iteratively, and given the nature of the issues, the mixed-methods and interdisciplinary approach was a key strength. However, the flexibility was also a disadvantage that limited generalisability of the results to some extent. The scope of the project was to identify the source of the odour so that it could be removed or managed more effectively, which narrowed the design. Ideally, the risk associated with the odour would have been assessed more comprehensively through exposure assessment and risk characterisation, but achieving this accurately was not realistic. As negative health effects had not been reported, the risk was ostensibly very low, and the driver for the research was financial—which is very often the scenario when working with industry.

Ideally, accurate identification and quantification of a compound from air samples would have been made, but this need was discounted based on the results of prior broad-spectrum sampling by commercial occupational hygienists, identified during the initial document review/gap analysis part of the research design. These reports did not identify any specific volatile compounds likely to be the cause of the odour. Additionally, air sampling after the laboratory simulation specifically to identify the presence or absence of TMA was not possible within the timeframes of the study. The remit was to complete the investigation well before the onset of summer, to allow mitigation methods to be applied before the climatic conditions conducive to the odour returned. As a result, it was highly unlikely that the odour would occur during the period of the investigation at a time when the researchers were present on site to collect samples. The global pandemic also restricted access to the affected trains, and logistics did not permit a comprehensive sampling of the trains after a strip down as would have been preferable. As a result, the decision was made to attempt to recreate the reported odour using insulation samples from implicated trains. This was based on a hypothesis that (1) the identified degraded seals would permit the ingress of moisture during the rainy conditions when the odour was reported; and (2) document analysis and discussion with the train manufacturer had identified that wet insulation was known to produce objectionable odours.

### Future research directions

The limitations of the action research design driving this study and the questions arising from the methodology pave the way for a number of other avenues of future research. For instance, in terms of the insulation foam material assessment, an increase of 2 pH units was a significant change in the chemistry of the surface and suggested that the compounds causing the odour and basicity may have had strong potency and/or exist in high concentrations. Should the odour phenomenon recur, this is something that would be useful to test in further work, together with a compete chemical fingerprinting of the ambient air and liquid on the insulation material surface to establish changes at the molecular level. The liquid material emanating from interstitial layers within the insulation material also needs to be chemically examined to identify its contents as this may pinpoint the exact contributors to the odours being observed. With the working hypothesis that the odour was due to TMA, gas chromatographic analysis would be the next step towards a conclusive outcome. This work also sets the foundation for a deeper quantitative study of additional aspects including chemical characterisation, release intensity and any risk to human health. Such work would be a useful addition to the body of knowledge amidst public health concerns around transport safety. Legislation based on such informed framework can help in minimising air pollution risks via preventative measurement and requirements of controlling such pollution [73].

## Conclusions

High numbers of microbial and fungal colonies are present within HVAC system components on trains, creating a potential to cause serious illness among those exposed to recircling air drawn through these components. Water ingress is a large driver of objectional odours reported by train operators and commuters and appear to be caused by volatile organic compounds released from wet insulation material after it dries out on particularly hot days. Given the likelihood of ongoing problems from recurrent water intrusion, measures need to be taken to prevent water ingress into the HVAC system and foam insulation in the first place. A frequent and detailed cleaning regime may help to address the odour phenomenon and minimise risk of issues more serious than just uncomfortable odours. However, while deep and more regular cleaning after water ingress may improve the sanitation of the HVAC and its components, release of hydrophilic chemicals from the materials into the water that has intruded is difficult to prevent. Instead, more frequent and robust inspections of HVAC components on trains would be warranted so material fatigue is identified in time for it to be addressed. Hindsight experience of the transmission of respiratory diseases (i.e., COVID-19) suggests that not addressing HVAC weaknesses may lead to further, more severe problems for public transport operators. More public health/environmental health awareness around the dangers of breathing recycled air may also be warranted. Closer re-examination of all known methods of viral transmission provoked by COVID-19 has presented a global opportunity to address all potential weaknesses through critical infrastructure (such as HVAC systems). However, a paradigm shift may be necessary to ensure that trains, HVACs, and their components, remain safe for the traveling public to use—both in terms of public health safety and comfort.

## Data Availability

Due to confidentiality agreements, neither the data nor the source of the data can be made available.
